# Machine learning and XAI approaches highlight the strong connection between $O_3$ and $NO_2$ pollutants and Alzheimer’s disease

**DOI:** 10.1038/s41598-024-55439-1

**Published:** 2024-03-05

**Authors:** Alessandro Fania, Alfonso Monaco, Nicola Amoroso, Loredana Bellantuono, Roberto Cazzolla Gatti, Najada Firza, Antonio Lacalamita, Ester Pantaleo, Sabina Tangaro, Alena Velichevskaya, Roberto Bellotti

**Affiliations:** 1 Dipartimento Interateneo di Fisica M. Merlin, Universitá degli Studi di Bari Aldo Moro, 70125 Bari, Italy; 2 Istituto Nazionale di Fisica Nucleare (INFN), Sezione di Bari, 70125 Bari, Italy; 3 Dipartimento di Farmacia - Scienze del Farmaco, Università degli Studi di Bari Aldo Moro, 70125 Bari, Italy; 4 Dipartimento di Biomedicina Traslazionale e Neuroscienze (DiBraiN), Università degli Studi di Bari Aldo Moro, 70124 Bari, Italy; 5 Department of Biological Sciences, Geological and Environmental (BiGeA), Alma Mater Studiorum - University of Bologna, 40126 Bologna, Italy; 6 Dipartimento di Economia e Finanza, Università degli Studi di Bari Aldo Moro, 70124 Bari, Italy; 7 Catholic University Our Lady of Good Counsel, 1031 Tirana, Albania; 8 Dipartimento di Scienze del Suolo, della Pianta e degli Alimenti, Università degli Studi di Bari Aldo Moro, 70126 Bari, Italy; 9 Biological Institute Tomsk State University, Tomsk, Russia 634050

**Keywords:** Alzheimer, Machine learning, Explainable artificial intelligence, Pollution, Standardized mortality ratio (SMR), One health, Neuroscience, Health care, Risk factors, Scientific data, Statistics, Neuroscience, Health care, Risk factors, Scientific data, Statistics

## Abstract

Alzheimer’s disease (AD) is the most common type of dementia with millions of affected patients worldwide. Currently, there is still no cure and AD is often diagnosed long time after onset because there is no clear diagnosis. Thus, it is essential to study the physiology and pathogenesis of AD, investigating the risk factors that could be strongly connected to the disease onset. Despite AD, like other complex diseases, is the result of the combination of several factors, there is emerging agreement that environmental pollution should play a pivotal role in the causes of disease. In this work, we implemented an Artificial Intelligence model to predict AD mortality, expressed as Standardized Mortality Ratio, at Italian provincial level over 5 years. We employed a set of publicly available variables concerning pollution, health, society and economy to feed a Random Forest algorithm. Using methods based on eXplainable Artificial Intelligence (XAI) we found that air pollution (mainly $O_3$ and $NO_2$) contribute the most to AD mortality prediction. These results could help to shed light on the etiology of Alzheimer’s disease and to confirm the urgent need to further investigate the relationship between the environment and the disease.

## Introduction

Alzheimer’s disease (AD) is the most common form of dementia currently affecting over 44 million people worldwide^1^. It is estimated that every 3 seconds, someone in the world develops dementia.

AD is characterized by a progressive deterioration of intellectual abilities. During the development of disease, the patient progressively loses intellectual faculties, initially regaining short-term memory, and then also including long-term memory and motor skills^2^.

The first symptoms, however, are generally revealed from the age of 50, with an increase in the incidence of the disease of about twice every 5 years, starting from 1 % from 64 years, up to 40 % for patients over 85 years^3^.

At the morphological level, the peculiarity of AD is an accumulation of proteins and specific neuropathological findings in the cortex area, in particular senile (neuritic) plaques and neurofibrillary clusters. The study of AD and the consequent creation of a care model appears very complex for a number of reasons: (i) the first symptoms of the disease can occur even many years after the onset; (ii) a definite diagnosis can only be made after death; (iii) the underlying causes of the disease could be many and not easy to identify. Therefore, given the multifactorial etiology of AD, a multidisciplinary “One Health” approach based on heterogeneous data linked to AD such as environmental, social, clinical factors could clarify the causes and the pathogenesis of dementia.

Although the etiology and pathogenesis of AD is not fully understood, oxidative stress is a key component^4–7^. The increase in oxidative stress could be translated into a greater risk of AD. Oxidative stress is a state of imbalance between the production and elimination of Reactive Oxygen Species (ROS) by antioxidant defense systems^8^. Reactive oxygen species are reactive molecules that present oxygen, therefore capable of oxidizing cells and important for maintaining oxygen homeostasis in tissues and destroying microbacteria^9^. Examples of ROS are the free radicals of oxygen and nitrogen (ROS and RNS) and their derivatives. The oxidation of lipids, proteins and DNA due to excessive amounts of ROS leads to deterioration of brain functions, including motor skills and it is responsible for the brain aging^10,11^.

In recent years, air pollution has been considered a major environmental risk factor for dementia^12–14^. The presence of ROS in the atmosphere seems to be connected to a common feature among AD patients: the olfactory dysfunction^15^. This dysfunction is due to the formation of neurofibrillary tangles in the olfactory bulb and olfactory centers before deposition in the brain. Air pollutants can directly act as prooxidants, favoring the production of free radicals, inducing inflammatory responses and oxidative stress^16^ and thus contributing to the onset and development of AD.

Reference^17^ highlights the hypothesis that Alzheimer’s and Parkinson’s diseases may be catalyzed by agents entering the brain via the olfactory mucosa.

Exposures to low-ozone doses are associated with neurodegenerative diseases because they causes a chronic oxidative stress state^18^. Ozone is the most widespread air pollutant and a powerful ROS agent produced by photochemical reactions between *NOX* and Volatile Organic Compounds (*VOC*) in the troposphere^19^. It can cause serious health problems in urban areas, especially during heavily polluted and strong sunlight days^20^.

Other studies reported that air pollution exposures, in particular $PM_{10}$ and $NO_2$^21,22^, may induce AD-like cortical atrophy and lead to poorer cognitive function^23–26^.

Not only air pollution but also soil pollution seems to be connected to the onset of Alzheimer. In fact pesticide use could increase the risk of developing AD^27–30^.

As evidence of the multifactorial nature of AD, also social factor could be considered in the prediction and prevention of AD. In particular some studies reported that low education is closely linked with increased cognitive decline or dementia^31–33^.^34^ reported that early life education can prevent the development of cognitive decline. Other social factors like life experiences and demographic influences showed an important role in incidence of dementia in adults^35^.

In our study, we aimed to investigate the connections between AD mortality, socio-economic factors, some clinical comorbidities and sources of environmental pollution in Italy at a provincial scale through an artificial intelligence approach. For this purpose we used publicly available indicators over the period 2015-2019 collected from national and regional agencies and computed the AD deaths incidence expressed as Standardized Mortality Ratio (SMR). We predicted the SMR by means of a Random Forest algorithm and investigated the role of each considered features in determining dementia mortality in each Italian provinces at different years through two different feature importance approaches: (i) a global one based on Random Forest; (ii) a local one by means of eXplainable Artificial Intelligence (XAI). The application of XAI techniques has allowed us to increase the transparency and interpretation of our machine learning model and to identify the features that gave a main contribute to dementia mortality.

## Materials and methods

**Figure 1:**
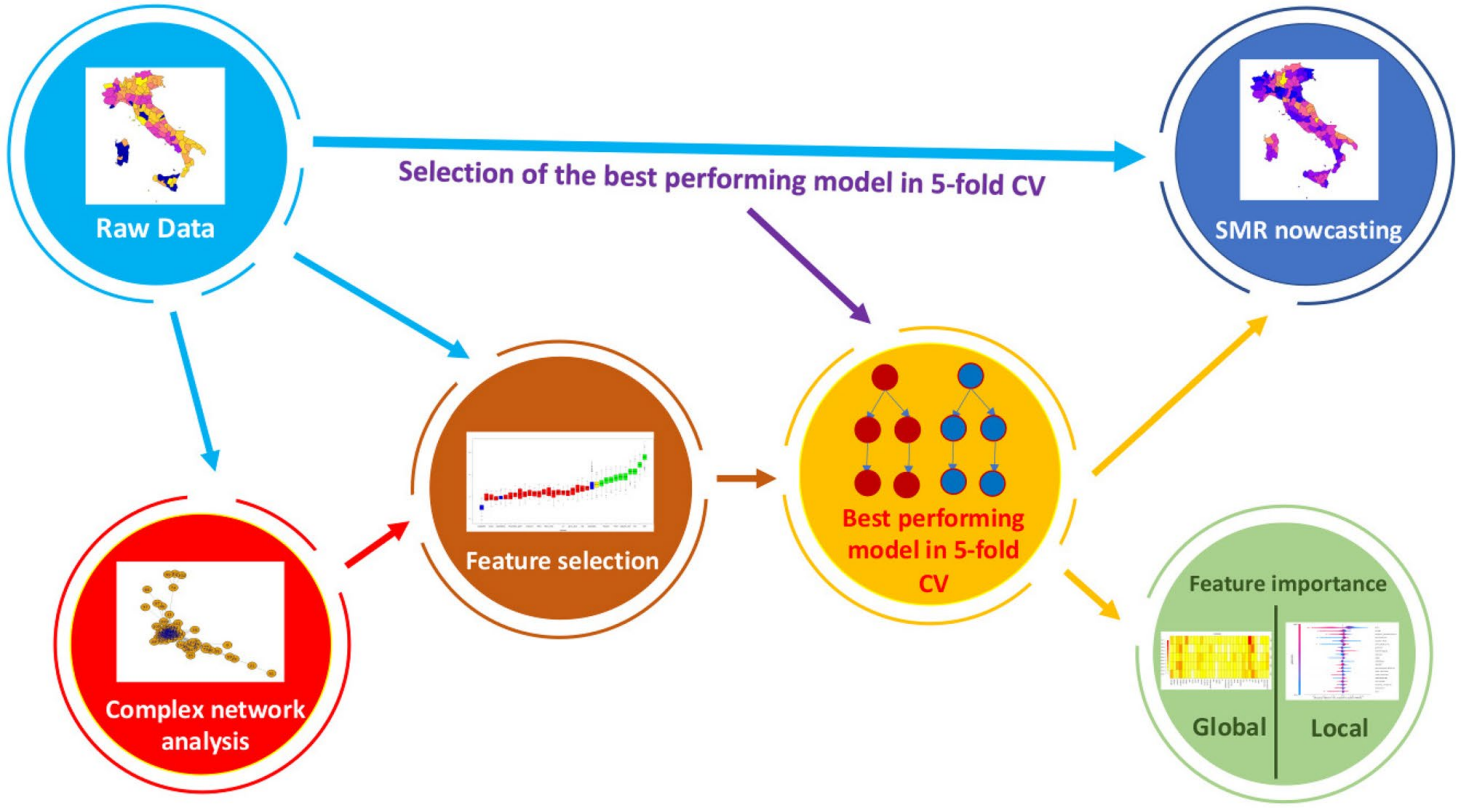
Flowchart of the implemented analysis. After a data collection and a pre-processing phase, we selected the best model (among LM and RF) to forecast the SMR for the 107 Italian provinces between 2015 and 2019. Then, we developed a feature importance procedure to improve the performance of the selected algorithm and to measure the importance of each variable in the model.

The main goal of our work was the investigation of possible significant correlations between different type of environmental pollution, socio-economic factors, clinical comorbidities and AD mortality in the Italian provinces from 2015 to 2019 through a procedure based on machine learning techniques. Our pipeline is summarized in Fig. 1.

To select the most performing algorithms between Linear Model (LM), and Random Forest (RF) we applied a common five-fold cross validation procedure. Then we used the feature set selected by wrapper method Boruta to feed this learning model for the prediction of SMR. Also to verify the robustness of our results we employed a complex networks tool used in previous works to analyze co-expression networks. Finally, we applied a feature importance procedure to outline the role of each feature at wider global and finer local scales with Random Forest internal functionalities and Shapley (SHAP) values algorithm respectively.

### Data collection and preprocessing

In this work we considered 31 indicators that we grouped into 5 categories: Air Pollution, Soil Pollution, Urban Environment, Socio-economic Data and Other Pathologies. The full list of such input variables, along with descriptions and related data sources, is reported in the Supplementary Information. We collected data for the years 2015-2019 of each Italian province from public repositories of Italian National Institute of Statistics (ISTAT) and Regional Environmental Protection Agencies (ARPAs). Only the feature “Life Quality Index” is provided by “Il Sole 24 Ore”. This index is calculated considering 90 different indicators. As for the clinical comorbidities we chose the mortality rate of some diseases connected to AD according to several studies, namely diabetes, ischemia, pathologies related to the circulatory, digestive and brain systems. Among Air Pollution data we also considered Air Quality Index (AQI) provided by http://moniqa.dii.unipi.it/. This index is obtained by dividing the measurement of the pollutant, by its reference limit, established by the Italian Legislative Decree 155/2010^36^.

All features used in this work are available at https://github.com/OneHealthBari/Italian-provinces-data.git

### Standardized AD mortality

ISTAT does not provide the Standardized Mortality Ratio (SMR) of AD for the 107 Italian provinces. Therefore, we computed it through the following definition^37,38^:

1 $$\begin{aligned} SMR = \frac{O_m}{E_m}, \end{aligned}$$

where $O_m$ and $E_m$ are the observed and the expected number of death by cause respectively. $E_m$ is defined as the weighted sum of age-specific death rates of the reference population $R_i^M$ per the population of a given locality and given age $n_i$:

2 $$\begin{aligned} E_m=\sum _{i=1}^{I} R_i^M \times n_i ,\end{aligned}$$

$R_i^M$ is obtained dividing the number of deaths by age and cause of the reference population $M_i$ with the age-specific reference population size $N_i$:

3 $$\begin{aligned} R_i^M=\frac{M_i}{N_i} ,\end{aligned}$$

**Figure 2:**
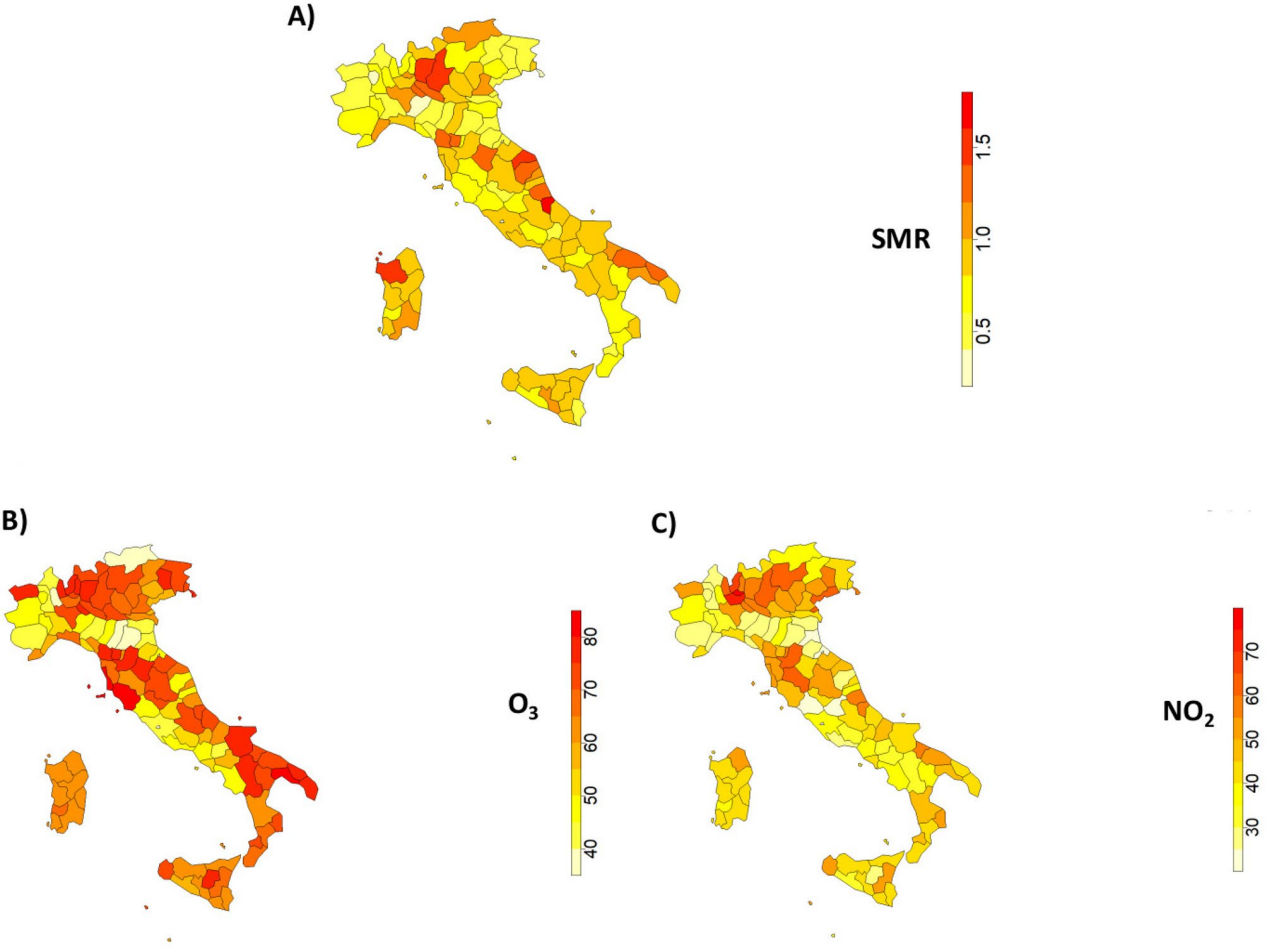
Panel (**A**) shows the average standardized mortality rate distribution for Alzheimer’s disease within Italian provinces; Panel (**B**) and Panel (**C**) show the average average distribution of $O_3$ and $NO_2$ concentrations ($\mu g/m^3$) at Italian provincial level. This image has been created with the software package “sf” of R 4.2.2^41^.

For a given province a SMR value higher than 1, means that the mortality incidence exceeds the reference one (we considered the Italian value as reference). Figure 2 shows the distribution of the value of the SMR (Panel A) and the concentrations of $O_3$ (Panel B) and $NO_2$ (Panel B) by province, averaged for the considered time window (2015–2019). Our computation of SMR is available on^39^ . More information about SMR computation is reported on^40^.

The study of AD is a very complex issue to deal with in terms of time, in fact we do not know exactly when the disease started, the onset could even go back decades before the patient’s death. Therefore, due to the peculiarity of the analyzed pathology and the impossibility of predicting the exact moment in which it began to develop, in our analysis we focused exclusively on the spatial characterization of the data. The idea is therefore to eliminate the temporal component from the analysis, and to study the spatial correlations between SMR and pollution, socio-economic and health data. We assumed that there have been no substantial changes in the spatial distributions of input data over the considered years.

### Feature selection procedure

We applied a feature selection procedure relied on the wrapper method Boruta^42^ with the aim to improve the performance of our selected learning model. This represents a common and extensively applied strategy in machine learning analysis. It involves an initial step of selecting features that optimize model performance using a wrapper algorithm. Subsequently, only the most informative features are fed into the algorithm as input to reduce noise. This approach helps to mitigate the potential pitfalls associated with standard machine learning applications, such as overfitting and underfitting. Boruta is a robust method, based on Random Forest, that reduces noise and correlated features through the randomization of the training set. For each original feature, the algorithm produces a synthetic one, called shadow, built by randomly mixing the values of each original indicator. With the new dataset (original features and shadows) Boruta trains a RF algorithm in order to compute, after a number of independent shuffles, the importance of both original and artificial variables. Finally only the features that are statistically more important than their respective shadow counterparts are selected. In particular, a permutation procedure is used to validate the role that the Random Forest (RF) algorithm assigns to the features and thus increase the robustness of the method. Shadow attributes are used as reference values to determine the importance of the features. When synthetic features have an importance that closely matches the corresponding best shadow features, it is challenging for Boruta to make decisions with the desired confidence. Boruta is intentionally designed to select all features that are relevant to the prediction of the outcome variable and that minimise prediction error. The specific steps that Boruta performs are as follows^43^:

- Permute each feature $X_j$ to create a shadow feature $X_j^{(s)}$.
- Build a Random Forest model using both the original and shadow features.
- Calculate the importance of each feature $X_j$ and $X_j^{(s)}$ by Mean Decrease Accuracy. Z-score is then calculated from the ratio between the mean accuracy loss and the standard deviation of the same distribution.
- Identify of the maximum Z-score among the shadow attributes (MZSA).
- Declare $X_j$ as important for a single run if its Z score exceeds the Z score of MZSA.
- Perform a two-sided statistical test for all attributes assuming the null hypothesis that the importance of the variables is equal to the maximum importance of MZSA. For each characteristic $X_j$, the algorithm records how often in *M* runs the importance of $X_j$ exceeds the MZSA (a hit is registered for the variable). The expected number of hits, following a binomial distribution with $p =$ $q =$ 0.5, is $E(M) = 0.5M$ with a standard deviation $S = \sqrt{0.25M}$. Subsequently, $X_j$ is categorized as important when the number of hits significantly exceeds *E*(*M*) and as unimportant when the number of hits significantly falls below *E*(*M*).
- Repeat the preceding steps for a predetermined number of iterations, or continue the process until all attributes are appropriately tagged.

The entire feature selection procedure was carried out within a 5-fold cross-validation framework as described below.

### Learning framework

We implemented a learning framework to forecast the SMR at provincial level. We fed our models with the features selected by Boruta. We started with a linear hypothesis and then we used a machine learning approach based on Random Forest (RF)^44^ to improve the model performances. Multiple linear regression is one of the most basic and used statistical models. This model investigates, under a linear hypothesis, the relationship between a dependent variable (*y*), some independent variables ($x_i$) and their interactions:

4 $$\begin{aligned} y = \beta _0 + \beta _1x_1 + \beta _2x_2 +... \beta _nx_x + \eta, \end{aligned}$$

where $\beta _0$ is the intercept value, $\beta _i$ are the regression coefficients to estimate, $\eta$ is the model error and *n* i the number of features selected by Boruta.

RF is an algorithm composed by an ensemble of binary classification trees (CART). Ensemble learning refers to the methodology of utilizing multiple models that are trained on the same dataset. The final output is determined by averaging the results produced by each individual model. This approach aims to achieve a more potent and robust predictive or classification result by leveraging the diverse perspectives and strengths of multiple models^44^. RF is a supervised machine learning model widely used because is suitable for modeling multimodal data and easy of tuning with on only two parameters to set: the number of randomly selected features at each node *F* , and the number of trees of the forest *D*. Furthermore RF is very robust against overfitting issue thanks to a training phase based on a bootstrap process and a feature randomization procedure during which the forest is developed. Thanks to the use of decision trees, the Random Forest (RF) algorithm is able to capture non-linear relationships present in the input features unlike the linear model. Another important functionality of RF is the ability to assess the importance of each variable used in the model through an internal feature importance procedure. In our work we trained RF model with a dataset composed by 107 Italian provinces and 31 socio-economic, health and pollution indicators. Furthermore, we used the mean decrease impurity as feature importance method, and a RF configurations with *M* = 600 trees and *F* = *S*/3, where S is the number of input features.

We applied a 5-fold cross validation (CV) framework, repeated 100 times, to further increase the robustness of our procedure. In the same way, RF overall feature importance is computed by averaging over 100 CVs.

We evaluated the performance of models using both the linear correlation coefficient between predicted and actual values and the root mean absolute error (MAE), defined as:

5 $$\begin{aligned} MAE =\frac{1}{n} \sum _{i=1}^{N} \left| A_i - P_i\right|, \end{aligned}$$

where $A_i$ is the actual value and $P_i$ is the predicted value.

### Complex networks tool

To verify the robustness of our findings we implemented a complex network approach. A complex network is a geometric model consisting of points (nodes) and lines (links) that symbolise the relationships between the elements within a complex system. The complex network approach is widely used in the study of complex systems because it provides information about the behaviour of the system through the abstraction of the network structure.

Our aim was to evaluate whether adding further confounding features to the ML model described in Sect. "Learning framework", our results remain constant. To do this we have created the network of Italian provinces. Starting from the dataset described in Sect. "Data collection and preprocessing" (107 provinces and 31 independent indicators) we build a network in which the nodes are represented by the Italian provinces. From this network, firstly we extracted some network features (4 new variables), able to capture the connections between different provinces and to provide additional spatial information to the model. Then we added the new features to the 31 indicators previously used and repeated the Machine Learning procedure described in the previous section.

In order to create the adjacency matrix we computed Spearaman’s correlation between each province, which is defined by an array of the 31 features described in Sect. "Data collection and preprocessing". We used the Spearman’s correlation for two main reasons: (i) outliers were present in analyzed dataset^45^; the sample size was quite small^46^. Given provinces *i* and *j*, we computed $d_{i,j}$, an element of the Spearman correlation matrix *D*, as the absolute value of the correlation coefficient $r_{i,j}$ between the indicator values of province *i* ($x_i$) and province *j* ($x_j$), for the $N = 31$ indicators:

6 $$\begin{aligned} d_{i,j}=|r_{i,j}|=\left| \frac{\sum _{a=1}^{N} (R(x_{i,a}) - R(\bar{x}_i))\sum _{b=1}^{N} (R(x_{j,b}) - R(\bar{x}_j))}{\sqrt{\sum _{a=1}^{N} (R(x_{i,a}) - R(\bar{x}_i))^2\sum _{b=1}^{N} (R(x_{j,b}) - R(\bar{x}_j))^2}}\right|, \end{aligned}$$

where $\bar{x}_i$ and $\bar{x}_j$ are the mean province *i* and province *j* indicator values across all the used indicators and $R(x_{i,a})$ the rank variable.

#### Adjacency matrix and information entropy

Starting from the correlation matrix *D* with the elements $d_{i,j}$, we computed the adjacency matrix *C* of elements $c_{i,j}$ by means of a hard thresholding procedure:

7 $$\begin{aligned} c_{i,j}={\left\{ \begin{array}{ll} 1, &{} \text {if} \,d_{i,j}\ge th \,\text {and} \,i\ne \,j, \\ 0, &{} \text {otherwise}, \end{array}\right. } \end{aligned}$$

where *th* indicates the optimal threshold that we selected to maximize the Shannon entropy based on Freeman’s betweenness centrality, a topological property of the network. So, we choose the network configuration that maximizes the entropy of betweenness distribution. The betweenness of node (province) *i* in a network with *G* elements (provinces) is defined as:

8 $$\begin{aligned} b_i=\sum _{i\ne j\ne k}^{G}\frac{n_{jk}(i)}{n_{jk}}. \end{aligned}$$

where $n_{jk}(i)$ is the number of geodesics (the shortest path connecting two nodes) between node *i* and node *j* that pass trough node *i* and $n_{jk}$ is the number of geodesics between node *j* and node *k*.

For each value of *th*, we got a different adjacency matrices $C^{(th)}$ and compared the Shannon entropy:

9 $$\begin{aligned} H_{C^{(th)}}=-\sum _{i=1}^G b_i^{(th)} \log _2(b_i^{(th)}) \end{aligned}$$

where $b_i^{(th)}$ is the betweenness of node (province) *i* in the network defined by adjacency matrix $C^{(th)}$.

The hard threshold analysis implemented in this work is an approach proposed in previous articles for the study of gene co-expression networks^47–49^.

#### Network centrality features

We calculated some quantities related to the intensity of the node connections and the weight distribution in order to be able to study the same nodes as their interaction changes. For each province in the network we computed the betweenness and the degree defined as the amount of connections of the node i:

10 $$\begin{aligned} k_{i}=\sum _{j=1}^{G}c_{ij}, \end{aligned}$$

Along with these two measurements we also evaluated the eigenvector centrality that quantifies a node’s importance while giving consideration to the importance of its neighbors and the closeness centrality of province i in a network with N nodes:

11 $$\begin{aligned} cl_{i}=\frac{N-1}{\sum _{j=1}^{N-1}s_{ij}}, \end{aligned}$$

where $s_{ij}$ is the shortest-path distance between i and j.

### Explainable artificial intelligence and Shapley values

The main purpose of Explainable Artificial Intelligence (XAI) is to increase transparency and interpretability of Machine and Deep Learning methods^50–52^. XAI refers to a set of techniques that combines a number of properties of AI models such as informativeness, uncertainty estimation, generalization and transparency^53,54^. In our analysis, we implemented the SHAP local explanation method to evaluate the role of each feature in the Random Forest model. Unlike the feature importance evaluated entirely by Random Forest which provides global information of the machine learning algorithm on the whole training set, SHAP gives the contribution of each feature in the prediction of the single observation. The SHAP algorithm is based on cooperative game theory^55,56^ and the concept of the Shapley (SHAP) values. Given all possible feature subsets *F* of the total feature set *S* ($F \subseteq S$) for a feature *j* the SHAP value is evaluated as the difference between two model outputs, the first obtained including that specific specific feature, the second without. The SHAP value of the j-*th* feature for the observation x is measured through the addition of the j-*th* feature to all possible subsets,

12 $$\begin{aligned} SHAP_j(x) = \sum _{F\subseteq S - \{j\}} \frac{|F|!(|S|-|F|-1)!}{|S|!} [f_{x}(F \cup {j})-f_{x}(F)], \end{aligned}$$

where |*F*|! is the number of feature permutations which precede the j-th feature; $(|S|-|F|-1)!$ is the number of feature permutations that follow the j-*th* feature value; |*S*|! represents the total number of permutations of features; $f_{x}(F)$ indicates the model prediction *f* for the sample *x*, considered a subset *F* without the j-*th* feature; $f_{x}(F \cup {j})$ is the output of the same model including the *j*-th feature^55^. In our analysis we computed the mean SHAP values after a 5-fold CV, repeated 100 times for each considered year.

Data processing and statistical analyses were performed in R 4.2.2^41^ and Python 3.9.

## Results

Firstly, we applied a preprocessing procedure, in which we filled the missing values in the considered dataset with the mean values of the corresponding features. Often a certain characteristic had no values for some provinces. Therefore, these missing values were replaced by the average value of the feature for the reference year. As suggested by Zhongheng Zhang^57^, the use of an estimate such as the mean for imputing missing values is appropriate in analyses with a limited number of samples, such as our study. Then we applied a linear model on the whole feature sample inside a repeated (100 times) 5-fold cross-validation framework (described in Sect. "Learning framework") for each considered year.

We used linear model results as benchmarks of our analysis. To improve the findings of linear model we implemented a RF classifier, compared the performances of the two models and selected the best performing one (RF). Then we applied Boruta algorithm to choose the most informative set of feature with which to fed RF inside a 5-fold cross-validation framework.

**Figure 3:**
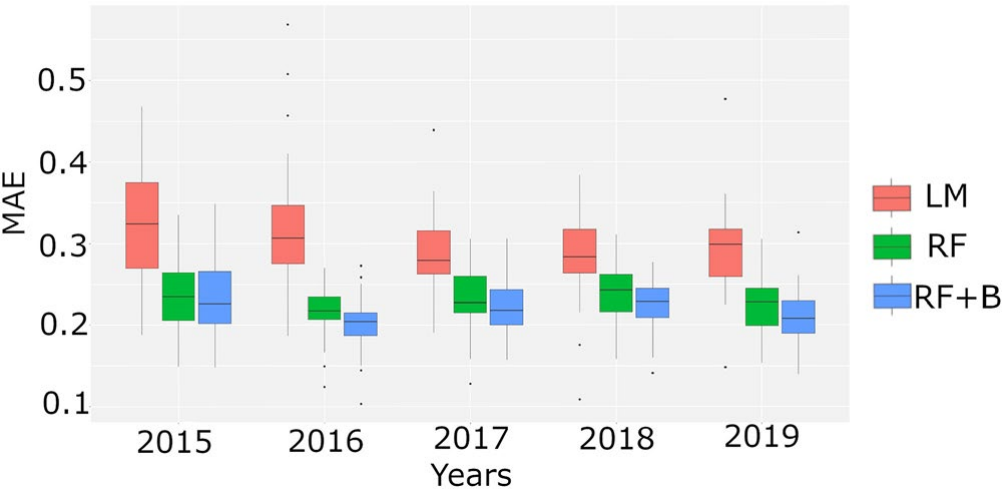
MAE for the three implemented models: linear model (LM), random forest (RF) and random forest with boruta (RF+B). Each distribution was computed through a 5-fold cross validation procedure repeated 100 times.

**Figure 4:**
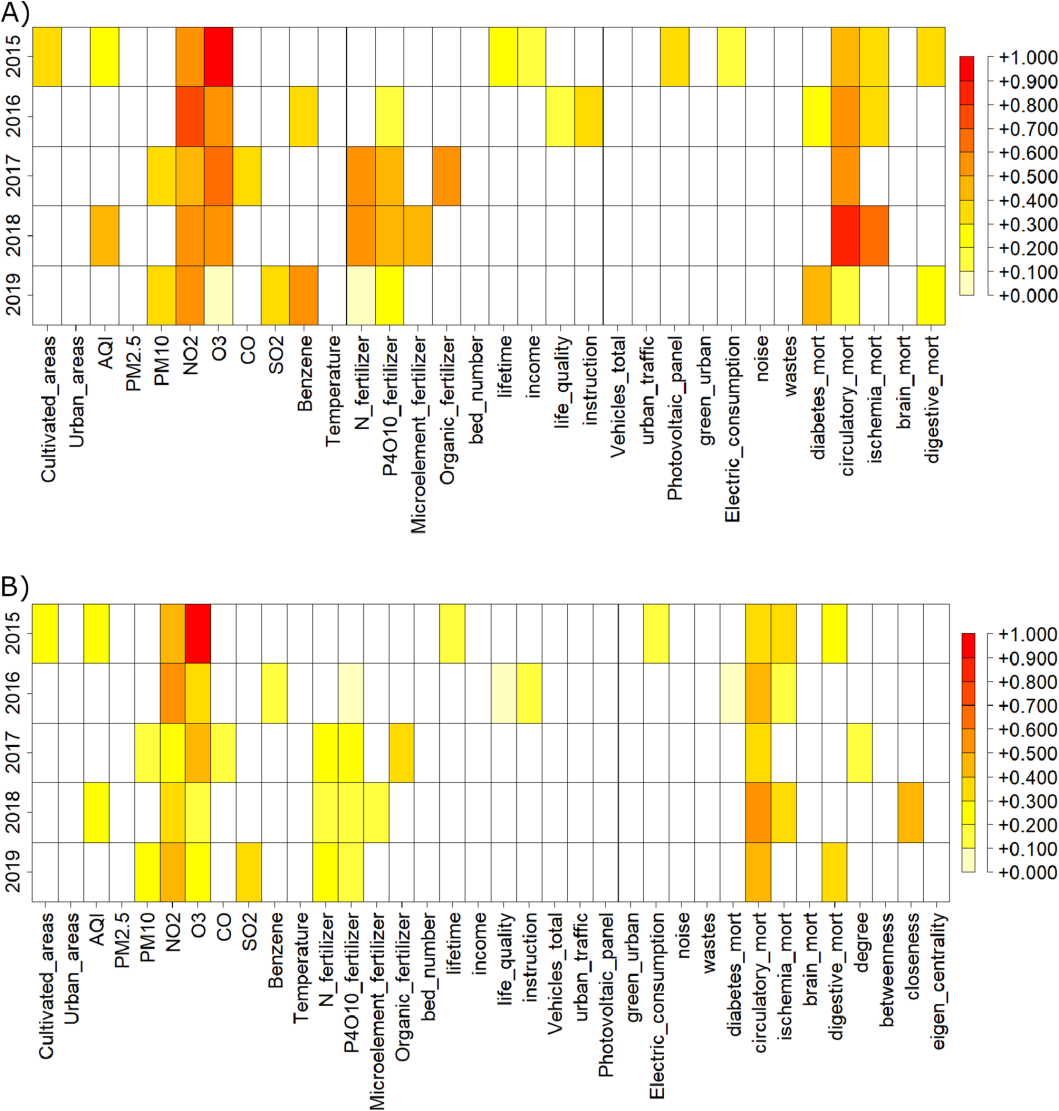
Average feature importance obtained through RF model after a 5-fold CV procedure with 100 ripetitions, for the considered time span (2015–2019). The white boxes show that the corresponding feature was rejected in the feature selection procedure relied on the Boruta algorithm. Panels (**A**) and (**B**) are referred to RF models trained with and without centrality network features respectively.

The performances obtained through the linear model, RF and RF fed by the Boruta features are reported in Table 1. All correlation coefficients of RF with and without Boruta are statistically significant at $1\%$. Figure 3 shows the distributions of MAE for each analyzed year: RF with Boruta resulted the best performing method. The results of the feature importance procedure assessed by means of RF algorithm are summarized in the panel A of Fig. 4, where the features importance for each year is shown in a color scale from red (high) to yellow (low). The features not selected by Boruta are indicated with white boxes. The analysis shows that $O_3$ and $NO_2$ have a consistently high importance.

Network analysis was then conducted. In particular we built the adjacency matrix through the hard thresholding procedure based on the Shannon entropy of betweenness described in Sect. Complex networks tool. Next, we computed the 4 network centrality metrics, added them to the original database, and repeated the machine learning and feature selection procedures for each year. The new RF performance shows no relevant changes, with a small difference of 0.1%. The role of the 4 network features is not relevant within the model as shown in panel B of the Fig. 4. The importance of the other features instead remains almost constant. Therefore, for the sake of simplicity, we performed SHAP analysis on the dataset without network metrics. We conclude that our results are robust even adding new features.

**Table 1:** Mean absolute error and p-values of the three developed models, for each year.

Year	Linear model		Random forest		Random forest + boruta	
	MAE	p-value	MAE	p-value	MAE	p-value
2015	0.32 ± 0.03	0.02 ± 0.03	0.24 ± 0.01	<1%	0.22 ± 0.01	<1%
2016	0.32 ± 0.03	0.03 ± 0.03	0.21 ± 0.01	<1%	0.20 ± 0.01	<1%
2017	0.29 ± 0.02	0.01 ± 0.01	0.23 ± 0.01	<1%	0.21 ± 0.01	<1%
2018	0.29 ± 0.02	0.01 ± 0.01	0.24 ± 0.01	<1%	0.23 ± 0.01	<1%
2019	0.30 ± 0.01	0.01 ± 0.01	0.22 ± 0.01	<1%	0.20 ± 0.01	<1%

All metrics are evaluated using a 5-fold Cross Validation repeated 100 times.

**Figure 5:**
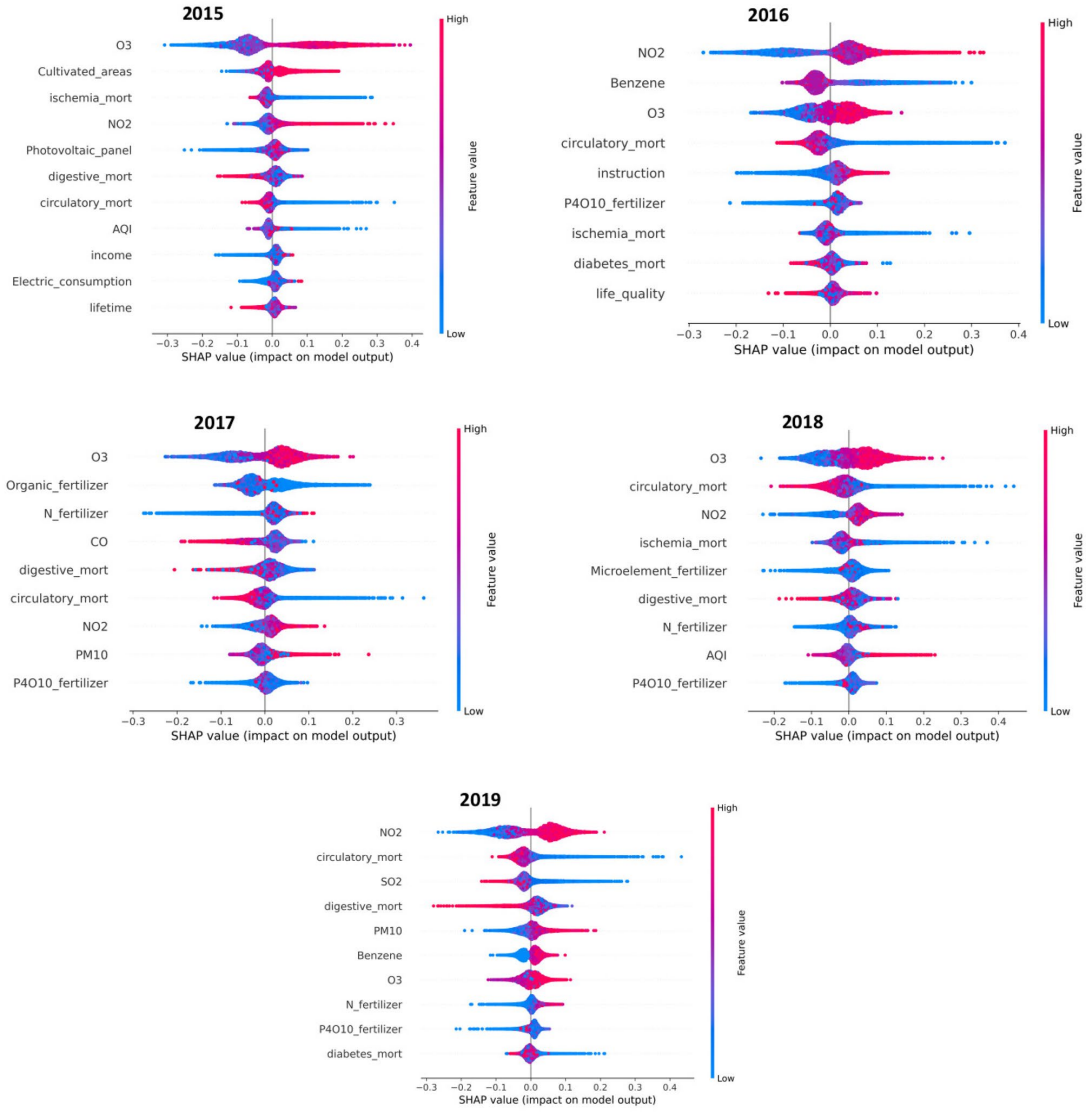
SHAP distribution values of the most influential features for the considered time window (2015–2019). Each point in the same row corresponds to a different province.

For each feature selected by Boruta and for each year, we computed the SHAP values associated to the SMR prediction. The means of these SHAP values have been reported in the Supplementary Information. Figure 5 show the distribution of SHAP values over the period 2015–2019 for each Italian province. These plots, where the features are ordered in terms of importance in the model, display that $O_3$, $NO_2$ always appear among the most important factors.

**Figure 6:**
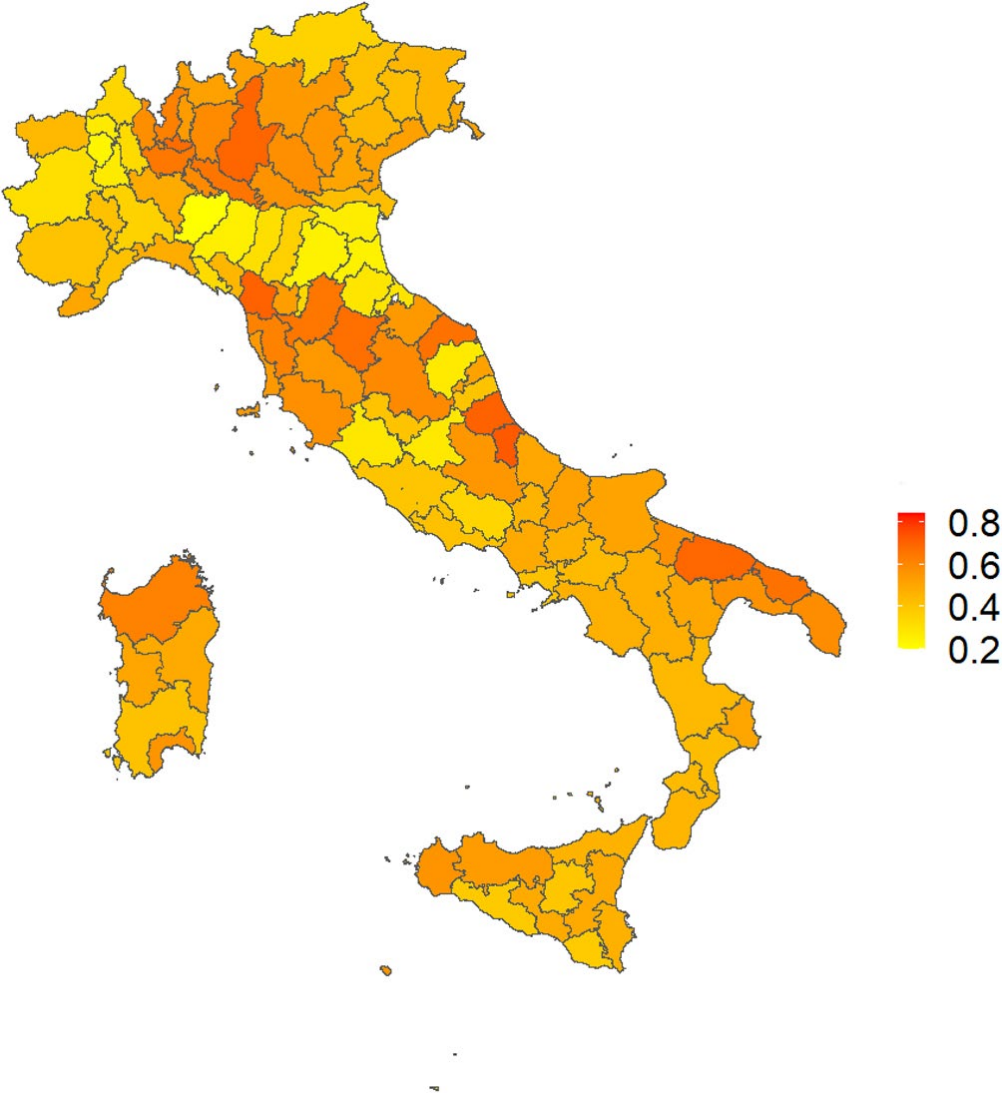
Average map between the SMR distribution and the $O_3$ and $NO_2$ concentrations shown in Fig. 2. Before averaging the 3 distributions, minimum maximum normalisation of the values was performed. This image has been created with the software package “sf” of R 4.2.2^41^.

Finally, to underline the differences in terms of AD mortality and pollution in the different provinces, we performed a min-max normalisation of both the SMR distribution and the $O_3$ and $NO_2$ concentrations shown in Fig. 2. From these three maps, we computed an average map, which is displayed in the Fig. 6.

## Discussion

In the present work, we focused on the relation between AD mortality and pollution, socio-economic and health indicators by means of a learning methodology based on Artificial Intelligence algorithms. We used 31 publicly available indicators to forecast the spatial distribution of AD deaths incidence expressed as Normalized Mortality Ratio (SMR) calculated for each Italian province over the period 2015-2019. First we chose the best performing method in terms of MAE and linear correlation between LM and RF. Then we selected the most important factors through the Boruta wrapper method and fed a Random Forest algorithm. Through RF with Boruta we obtained a good average performance of 0.21 of MAE over all considered years. To verify the robustness of our results we implemented a complex network approach, never applied on this type of data. Through a procedure based on Spearman’s correlation and Shannon’s entropy we built the network of the Italian provinces. From this network we extracted 4 features that we added to the 31 indicators previously used and we fed the machine learning model repeating the procedure described in Sect. "Learning framework". These measures of centrality reported in Sect. "Network centrality features" condense spatial information at various levels. The new features do not improve the performance of our procedure (we only noted a difference of 0.1%) but this procedure shown a good stability of our findings. In fact, the role of the most important features in the model remains unchanged even by adding factors that could be confounding. The result of the implemented global feature importance procedure based on Boruta and Random Forest, shown in Fig. 4, highlights the burden of environmental pollution on AD mortality. Overall, we found that $O_3$, $NO_2$ and the mortality rate due to diseases of circulatory system are among the most important factors associated with high SMR values. A clear spatial association between AD mortality and $O_3$ and $NO_2$ at a provincial scale emerges through a comparison among the average distribution (over 5 years) of SMR, shown in panel A of Fig. 2 and the average concentrations of $O_3$ and $NO_2$ shown in the panel B and C of the same Figure, confirming the existence of some geographic patterns. To emphasise these patterns, we calculated the average map of the maps shown in Fig. 2. The geographical areas with the most intense colour scale have the highest values for both pollution and SMR. It is evident that most of the provinces in Puglia, Abruzzo, Tuscany and Lombardy are significantly affected by both high AD mortality risk and relevant levels of $O_3$ and $NO_2$ pollutants, although they differ in geographical, meteorological and socio-economic terms. This confirms that AD mortality and air pollution are more associated to each other than any other variable that could explanation different patterns.

The pivotal role of $O_3$ and $NO_2$ in the model is confirmed also by XAI analysis displayed in Fig. 5. An important information provided by SHAP results is how the considered features influence the sign of the SHAP values. The behaviors of $O_3$ and $NO_2$ in the Shapley plots indicate that high concentrations of these air pollutants correspond to an excess of AD deaths. So both Boruta and SHAP highlighted the importance of $O_3$ and $NO_2$ pollutants, giving a global and local explanation respectively. As previous mentioned, these results are already investigated in several works. Studies on rats have shown that long-term exposure to ozone, produces short- and long-term memory loss, along with motor disabilities^18,58^.^59^ underlined the effect of chronic ozone exposure on brain tissue and the close relationship between ozone pollution and neurodegenerative diseases.^14^ reported an association among increase in ozone concentration in Rome and increase in risk of hospitalisation with dementia.^60^ found a high risk of AD in areas of Taiwan strongly polluted by ozone .^61^ reported evidence of a positive correlation among residential levels of air pollution across London (in particular $NO_2$) and people diagnosed with AD and vascular dementia. These findings are in agreement with many other works which conclude that increased $NO_2$ concentrations are linked to an elevated risk of AD (and other forms of dementia) incidence^62–64^.

As we have already said, another important finding underlined by our feature importance procedure is the relevant rule of mortality related to cardiovascular diseases in the prediction of SMR. SHAP analysis underlined that low mortality rate for the circulatory system problem correspond to high AD mortality values. This result is confirmed by a negative correlation coefficient ($-0.32$) between SMR and the feature “circulatory system”, but appears to be in contrast to the findings of other works in which AD and cardiovascular diseases are reported strongly associated^65,66^. An explanation of our results may be due to the type of data used since they are mortality rates and not incidence rates. The negative correlation between SMR for AD and the mortality rate for cardiovascular diseases is likely due to the fact that a large number of patients with dementia also suffer from heart problems, but death from cardiac causes precedes that from AD and vice versa. This strong association between AD and cardiovascular disease translates into an inverse association of the relative mortality rates. Many other work reported this link between AD and cardiovascular diseases.

In a study carried by Scherbakov and Doehner, it was showed that brain is subject to cerebral perfusion, which commonly happens in hearth failure. The reason is the high vascularization of the brain^67^.^68^ found that cerebral hypoperfusion increases the production of neurofibrillary tangles and Amyloid-$\beta$ (A$\beta$) plaques, characteristic of AD. Also, the hypoperfusion causes the breakdown of the blood-brain barrier, which obstructs the removal of A$\beta$^69^. Another mechanism which play an important role in the formation of amyloid plaques, in cases of hypoperfusion, is the breakdown of the blood-brain barrier, which impairs the clearance of A$\beta$^69^. To our knowledge, our work represents the most comprehensive study of the close connection between environmental factors (especially pollution) and dementia affecting an entire Italian population so far. Our analysis, through the support of machine learning and XAI techniques and with a large set of variables, provides a view of the most important factors both at a global and local level.

We acknowledge that our study has some limitations. The first one is the incipient nature of dementia that increases the difficulty of accurate diagnosis and proves problematic in detecting associations with AD risk factors. This aspect leads to a possible underestimation of AD mortality value. In fact, as we have already said, it is possible to diagnose AD only after the patient’s death, therefore the need to perform an autopsy automatically generates an underestimation of the mortality rate. Another limitation to this analysis is represented by the estimation of the concentrations of the analyzed pollutants. We assessed the concentration of air pollutants in a province through the available monitoring stations, which does not cover the whole territory. So we made a spatial approximation. A possible evolution of this approach consists in the creation of a pollution map of Italian territory by means of satellite data (for example Sentinel-5 mission) which have a large point density. Our results suggest that constant monitoring over the years and the ever-increasing availability of data will also help predict the evolution of AD in the future, given that it occurs many decades after exposure to pollutants.

## Conclusions

In this study we investigated possible risk factors of AD taking into account pollution socio-economic and health data. We built a model, based on Machine Learning and Explainable Artificial Intelligence techniques and complex networks framework to predict the AD mortality at the Italian provincial level over a period of 5 years (2015–2019). Our model presented a good precision with a mean absolute error of 0.22. Through a feature importance procedure relied on global and local approaches we found a link between pollution and AD with $O_3$ and $NO_2$ that assumed pivotal rules in the model. Although our analysis presents some limitations due to the physiology of AD, our findings are promising and deserve further investigation also by means of satellite data to estimate pollution concentrations.

### Supplementary Information

Supplementary Tables.

## Data Availability

The datasets of SMR generated and analysed during the current study are available in a Dryad data repository$^39$. The other datasets used and analysed during the current study are available from the corresponding author on reasonable request.

## References

[1] WHO. Dementia. https://www.who.int/news-room/fact-sheets/detail/dementia (2022).

[2] Shin, I. S., Carter, M., Masterman, D., Fairbanks, L. & Cummings, J. L. Neuropsychiatric symptoms and quality of life in Alzheimer disease. *Am. J. Geriatr. Psychiatry* **13**, 469–479 (2005). 15956266 10.1097/00019442-200506000-00005

[3] Kumar, V., Fausto, N. & Abbas, A. R. *Cotran Pathologic Basis of Disease* (Saunders, 2004).

[4] Markesbery, W. R. Oxidative stress hypothesis in Alzheimer’s disease. *Free Radic. Biol. Med.* **23**, 134–147 (1997). 9165306 10.1016/S0891-5849(96)00629-6

[5] Butterfield, D. & Halliwell, B. Oxidative stress, dysfunctional glucose metabolism and Alzheimer disease. *Nat. Rev. Neurosci.* **20**, 148–160 (2019). 30737462 10.1038/s41583-019-0132-6 PMC9382875

[6] Misrani, A., Tabassum, S. & Yang, L. Mitochondrial dysfunction and oxidative stress in Alzheimer’s disease. *Front. Aging Neurosci.* **13**, 617588 (2021). 33679375 10.3389/fnagi.2021.617588 PMC7930231

[7] Huang, W., Zhang, X. & Chen, W. Role of oxidative stress in Alzheimer’s disease. *Biomed. Rep.* **4**, 519–522 (2016). 27123241 10.3892/br.2016.630 PMC4840676

[8] Storz, G. & Imlayt, J. A. Oxidative stress. *Curr. Opin. Microbiol.* **2**, 188–194 (1999). 10322176 10.1016/S1369-5274(99)80033-2

[9] Ray, P. D., Huang, B.-W. & Tsuji, Y. Reactive oxygen species (ros) homeostasis and redox regulation in cellular signaling. *Cell. Signal.* **24**, 981–990 (2012). 22286106 10.1016/j.cellsig.2012.01.008 PMC3454471

[10] Del Valle, L. G. Oxidative stress in aging: Theoretical outcomes and clinical evidences in humans. *Biomed. Aging Pathol.* **1**, 1–7 (2011). 10.1016/j.biomag.2011.03.001 20950991

[11] Ionescu-Tucker, A. & Cotman, C. W. Emerging roles of oxidative stress in brain aging and Alzheimer’s disease. *Neurobiol. Aging* **107**, 86–95 (2021). 34416493 10.1016/j.neurobiolaging.2021.07.014

[12] Shi, L. *et al.* Long-term effects of pm(25) on neurological disorders in the American Medicare population: A longitudinal cohort study. *Lancet Planet Health* **4**, e557–e565 (2020). 33091388 10.1016/S2542-5196(20)30227-8 PMC7720425

[13] Delgado-Saborit, J. *et al.* A critical review of the epidemiological evidence of effects of air pollution on dementia, cognitive function and cognitive decline in adult population. *Sci. Total Environ.* **757**, 143734 (2021). 33340865 10.1016/j.scitotenv.2020.143734

[14] Cerza, F. *et al.* Long-term exposure to air pollution and hospitalization for dementia in the Rome longitudinal study. *Environ. Health* **18**, 72 (2019). 31399053 10.1186/s12940-019-0511-5 PMC6689157

[15] Silva, M. D. M., Mercer, P. B. S., Witt, M. C. Z. & Pessoa, R. R. Olfactory dysfunction in Alzheimer’s disease systematic review and meta-analysis. *Dement. Neuropsychol.* **12**, 123–132 (2018). 29988355 10.1590/1980-57642018dn12-020004 PMC6022986

[16] Becker, S., Soukup, J. & Gallagher, J. Differential particulate air pollution induced oxidant stress in human granulocytes, monocytes and alveolar macrophages. *Toxicol. In Vitro* **16**, 209–218 (2002). 12020593 10.1016/S0887-2333(02)00015-2

[17] Doty, R. L. The olfactory vector hypothesis of neurodegenerative disease: Is it viable?. *Ann. Neurol. Off. J. Am. Neurol. Assoc. Child Neurol. Soc.* **63**, 7–15 (2008). 10.1002/ana.21327 18232016

[18] Velázquez-Pérez, R. *et al.* Oxidative stress caused by ozone exposure induces changes in p2x7 receptors, neuroinflammation, and neurodegeneration in the rat hippocampus. *Oxid. Med. Cell. Longev.* **2021**, 3790477 (2021). 34790285 10.1155/2021/3790477 PMC8592727

[19] Monks, P. S. *et al.* Tropospheric ozone and its precursors from the urban to the global scale from air quality to short-lived climate forcer. *Atmos. Chem. Phys.* **15**, 8889–8973 (2015). 10.5194/acp-15-8889-2015

[20] Zhang, J., Wei, Y. & Fang, Z. Ozone pollution: A major health hazard worldwide. *Front. Immunol.* **10**, 2518 (2019). 31736954 10.3389/fimmu.2019.02518 PMC6834528

[21] Block, M. L. & Calderón-Garcidueñas, L. Air pollution: Mechanisms of neuroinflammation and CNS disease. *Trends Neurosci.* **32**, 506–516 (2009). 19716187 10.1016/j.tins.2009.05.009 PMC2743793

[22] Cho, J. *et al.* Alzheimer’s disease-like cortical atrophy mediates the effect of air pollution on global cognitive function. *Environ. Int.* **171**, 107703 (2023). 36563596 10.1016/j.envint.2022.107703

[23] Power, M. *et al.* The association of long-term exposure to particulate matter air pollution with brain MRI findings: The ARIC study. *Environ. Health Perspect.* **126**, 027009 (2018). 29467108 10.1289/EHP2152 PMC6066342

[24] Cho, J. *et al.* Long-term ambient air pollution exposures and brain imaging markers in Korean adults: The environmental pollution-induced neurological effects (epinef) study. *Environ. Health Perspect.* **128**, 117006 (2020). 33215932 10.1289/EHP7133 PMC7678746

[25] Chen, J. *et al.* Ambient air pollution and neurotoxicity on brain structure: Evidence from women’s health initiative memory study. *Ann. Neurol.* **78**, 466–476 (2015). 26075655 10.1002/ana.24460 PMC4546504

[26] Casanova, R. *et al.* A voxel-based morphometry study reveals local brain structural alterations associated with ambient fine particles in older women. *Front. Hum. Neurosci.* **10**, 49 (2016). 27790103 10.3389/fnhum.2016.00495 PMC5061768

[27] Sarailoo, M., Afshari, S., Asghariazar, V., Safarzadeh, E. & Dadkhah, M. Cognitive impairment and neurodegenerative diseases development associated with organophosphate pesticides exposure: A review study. *Neurotox Res.* **40**, 1624–1643 (2022). 36066747 10.1007/s12640-022-00552-0

[28] Sturm, E. *et al.* Risk factors for brain health in agricultural work: A systematic review. *Int. J. Environ. Res. Public Health* **19**, 3373 (2022). 35329061 10.3390/ijerph19063373 PMC8954905

[29] Zaganas, I. *et al.* Linking pesticide exposure and dementia: What is the evidence?. *Toxicology* **307**, 3–11 (2013). 23416173 10.1016/j.tox.2013.02.002

[30] Baldi, I. *et al.* Neurodegenerative diseases and exposure to pesticides in the elderly. *Am. J. Epidemiol.* **157**, 409–414 (2003). 12615605 10.1093/aje/kwf216

[31] Harrison, S. *et al.* Exploring strategies to operationalize cognitive reserve: A systematic review of reviews. *J. Clin. Exp. Neuropsychol.* **37**, 253–264 (2015). 25748936 10.1080/13803395.2014.1002759

[32] Takasugi, T. *et al.* Community-level educational attainment and dementia: A 6-year longitudinal multilevel study in Japan. *BMC Geriatr.* **21**, 1–10 (2021). 34814847 10.1186/s12877-021-02615-x PMC8609807

[33] Sharp, E. & Gatz, M. Relationship between education and dementia: An updated systematic review. *Alzheimer Dis. Assoc. Disord.* **24**, 289–304 (2011). 10.1097/WAD.0b013e318211c83c PMC3193875 21750453

[34] Zahodne, L. B., Stern, Y. & Manly, J. J. Differing effects of education on cognitive decline in diverse elders with low versus high educational attainment. *Neuropsychology* **29**, 649 (2015). 25222199 10.1037/neu0000141 PMC4362867

[35] Brewster, P. W. *et al.* Life experience and demographic influences on cognitive function in older adults. *Neuropsychology* **28**, 846 (2014). 24933483 10.1037/neu0000098 PMC4227962

[36] Cazzolla Gatti, R., Velichevskaya, A., Tateo, A., Amoroso, N. & Monaco, A. Machine learning reveals that prolonged exposure to air pollution is associated with sars-cov-2 mortality and infectivity in Italy. *Environ. Pollut.* **267**, 115471 (2020). 32882464 10.1016/j.envpol.2020.115471 PMC7442434

[37] Cazzolla Gatti, R. *et al.* A ten-year (2009–2018) database of cancer mortality rates in Italy. *Sci. Data* **9**, 638 (2022). 36270998 10.1038/s41597-022-01729-0 PMC9586951

[38] Cazzolla Gatti, R. *et al.* The spatial association between environmental pollution and long-term cancer mortality in Italy. *Sci. Total Environ.* **855**, 158439 (2023). 36113788 10.1016/j.scitotenv.2022.158439

[39] Fania, A. *et al.* Dementia mortality rates at the provincial level in Italy from 2012–2019 in the form of Standardized Mortality Ratios (SMR). *Dryad*10.5061/dryad.18931zd2m (2023). 10.5061/dryad.18931zd2m

[40] Fania, A. *et al.* A dementia mortality rates dataset in Italy (2012–2019). *Sci. Data* **10**, 564 (2023). 37626087 10.1038/s41597-023-02461-z PMC10457292

[41] R Core Team. R: A language and environment for statistical computing. R Foundation for Statistical Computing, Vienna, Austria. https://www.r-project.org/ (2018).

[42] Kursa, M. & Rudnicki, W. Feature selection with the Boruta package. *J. Stat. Softw.* **36**, 1–13. 10.18637/jss.v036.i11 (2010). 10.18637/jss.v036.i11

[43] Kursa, M. B., Jankowski, A. & Rudnicki, W. R. Boruta – a system for feature selection. *Fund. Inform.* **101**, 271–285 (2010).

[44] Breiman, L. Random forests. *Mach. Learn.* **45**, 32–45. 10.1023/A:1010933404324 (2001). 10.1023/A:1010933404324

[45] de Winter, J. C. F., Gosling, S. D. & Potter, J. Comparing the Pearson and Spearman correlation coefficients across distributions and sample sizes: A tutorial using simulations and empirical data. *Psychol. Methods* **21**, 273–290 (2016). 27213982 10.1037/met0000079

[46] Bonett, D. G. & Wright, T. A. Sample size requirements for estimating Pearson, Kendall and spearman correlations. *Psychometrika* **65**, 23–28 (2000). 10.1007/BF02294183

[47] Monaco, A. *et al.* A complex network approach reveals a pivotal substructure of genes linked to schizophrenia. *PLoS One* **13**, e0190110 (2018). 29304112 10.1371/journal.pone.0190110 PMC5755767

[48] Monaco, A. *et al.* Shannon entropy approach reveals relevant genes in Alzheimer’s disease. *PLoS One* **14**, e0226190 (2019). 31891941 10.1371/journal.pone.0226190 PMC6938408

[49] Monaco, A. *et al.* Identifying potential gene biomarkers for Parkinson’s disease through an information entropy based approach. *Phys. Biol.* **18**, 016003 (2020). 33049726 10.1088/1478-3975/abc09a

[50] Jiménez-Luna, J., Grisoni, F. & Schneider, G. Drug discovery with explainable artificial intelligence. *Nat. Mach. Intell.* **2**, 573–584 (2020). 10.1038/s42256-020-00236-4

[51] Miller, T. Explanation in artificial intelligence: Insights from the social sciences. *Artif. Intell.* **267**, 1–38 (2019). 10.1016/j.artint.2018.07.007

[52] Bussmann, N., Giudici, P., Marinelli, D. & Papenbrock, J. Explainable AI in fintech risk management. *Front. Artif. Intell.* **3**, 26 (2020). 33733145 10.3389/frai.2020.00026 PMC7861223

[53] Flach, P. Performance evaluation in machine learning: The good, the bad, the ugly, and the way forward. *Proc. AAAI Conf. Artif. Intell.* **33**, 9808–9814 (2019).

[54] Vollmer, S. *et al.* Machine learning and artificial intelligence research for patient benefit: 20 critical questions on transparency, replicability, ethics, and effectiveness. *BMJ* **368**, 16927 (2020). 10.1136/bmj.l6927 PMC11515850 32198138

[55] Lundberg, S. & Lee, S. A unified approach to interpreting model predictions. In *Proc. of the 31st International Conference on Neural Information Processing Systems*, 44768–4777 (2017).

[56] Lundberg, S. *et al.* From local explanations to global understanding with explainable ai for trees. *Nat. Mach. Intell.* **2**, 56–67 (2020). 32607472 10.1038/s42256-019-0138-9 PMC7326367

[57] Zhang, Z. Missing data imputation: Focusing on single imputation. *Ann. Transl. Med.* **4**, 9 (2016). 26855945 10.3978/j.issn.2305-5839.2015.12.38 PMC4716933

[58] Rivas-Arancibia, S. *et al.* Oxidative stress caused by ozone exposure induces loss of brain repair in the hippocampus of adult rats. *Toxicol. Sci.* **113**, 187–197 (2010). 19833740 10.1093/toxsci/kfp252

[59] Bello-Medina, P., Rodríguez-Martínez, E., Prado-Alcalá, R. & Rivas-Arancibia, S. Ozone pollution, oxidative stress, synaptic plasticity, and neurodegeneration. *Neurologia (Engl. Ed.)* **37**, 277–286 (2022). 34531154 10.1016/j.nrleng.2018.10.025

[60] Jung, C. & Lin, Y. Ozone, particulate matter, and newly diagnosed Alzheimer’s disease: A population-based cohort study in Taiwan. *J. Alzheimers Dis.* **44**, 573–84 (2015). 25310992 10.3233/JAD-140855

[61] Carey, I. *et al.* Are noise and air pollution related to the incidence of dementia? A cohort study in London, England. *BMJ Open* **8**, e022404 (2018). 30206085 10.1136/bmjopen-2018-022404 PMC6144407

[62] Chang, K.-H. *et al.* Increased risk of dementia in patients exposed to nitrogen dioxide and carbon monoxide: A population-based retrospective cohort study. *PLoS One* **9**, e103078 (2014). 25115939 10.1371/journal.pone.0103078 PMC4130523

[63] Li, C.-Y., Li, C.-H., Martini, S. & Hou, W.-H. Association between air pollution and risk of vascular dementia: A multipollutant analysis in Taiwan. *Environ. Int.* **133**, 105233 (2019). 31678904 10.1016/j.envint.2019.105233

[64] Smargiassi, A. *et al.* Exposure to ambient air pollutants and the onset of dementia in Québec, Canada. *Environ. Res.* **190**, 109870 (2020). 32739624 10.1016/j.envres.2020.109870

[65] Tini, G. *et al.* Alzheimer’s disease and cardiovascular disease: A particular association. *Cardiol. Res. Pract.* **2020**, 1–10 (2020). 10.1155/2020/2617970 PMC7222603 32454996

[66] Cho, S. *et al.* Association of cardiovascular health with the risk of dementia in older adults. *Sci. Rep.* **12**, 15673 (2022). 36123419 10.1038/s41598-022-20072-3 PMC9485258

[67] Scherbakov, N. & Doehner, W. Heart-brain interactions in heart failure. *Cardiac Fail. Rev.* **4**, 87 (2018). 10.15420/cfr.2018.14.2 PMC6125712 30206482

[68] Stampfer, M. Cardiovascular disease and Alzheimer’s disease: Common links. *J. Intern. Med.* **260**, 211–223 (2006). 16918818 10.1111/j.1365-2796.2006.01687.x

[69] Purnell, C., Gao, S., Callahan, C. M. & Hendrie, H. C. Cardiovascular risk factors and incident Alzheimer disease: A systematic review of the literature. *Alzheimer Dis. Assoc. Disord.* **23**, 1 (2009). 18703981 10.1097/WAD.0b013e318187541c PMC3689425

