# Supplementary material for: Machine learning and XAI approaches highlight the strong connection between O3 and NO2 pollutants and Alzheimer’s disease
Source: Sci Rep. 2024 Mar 5;14:5385. doi: 10.1038/s41598-024-55439-1 (PMC11319812; doi:10.1038/s41598-024-55439-1)
Supplement: Supplementary file 1 — Supplementary Tables. [file 41598_2024_55439_MOESM1_ESM.pdf]

1. Supplementary Materials

Name	Category	Description	Source	Feature Name
NO_2	Air Pollution	Density of NO_2 present in the air ($\mu g/m^3$).	Arpa	NO2
O_3	Air Pollution	Density of O_3 present in the air ($\mu g/m^3$).	Arpa	O3
SO_2	Air Pollution	Density of SO_2 present in the air ($\mu g/m^3$).	Arpa	SO2
$Benzene$	Air Pollution	Density of $Benzene$ present in the air ($\mu g/m^3$).	Arpa	Benzene
$PM2.5$	Air Pollution	Density of $PM_{2.5}$ present in the air ($\mu g/m^3$).	Arpa	PM2.5
$PM10$	Air Pollution	Density of PM_{10} present in the air ($\mu g/m^3$).	Arpa	PM10
CO	Air Pollution	Density of CO present in the air ($\mu g/m^3$).	Arpa	CO
AQI	Air Pollution	Index calculated using the average percentage of concentration of the main pollutants measured in an hour, eight hours or a whole day.	Arpa	AQI
Cultivated Areas	Soil Pollution	Percentage of Cultivated Areas over province. (%).	Istat	Cultivated_areas
Urban Areas	Soil Pollution	Percentage of Urban Areas over province. (%).	Istat	Urban_areas

N	Soil Pollution	Tons of N present in the fertilizers dispensed (kg).	Istat	N_fertilizer
P_4O_{10}	Soil Pollution	Tons of P_4O_{10} present in the fertilizers dispensed (kg).	Istat	P4O10_fertilizer
Microelements	Soil Pollution	Tons of *Microelements* present in the fertilizers dispensed (kg).	Istat	Microelement_fertilizer
Organic Supports	Soil Pollution	Tons of *Organic Supports* present in the fertilizers dispensed (kg).	Istat	Organic_fertilizer
Noise	Urban Environment	Number of exceedances of the limits detected as a result of noise controls carried out for all sources and for those service or commercial activities.	Istat	noise
Municipal Waste	Urban Environment	Collection of total municipal waste ($kg/inhabitant$).	Istat	wastes
Urban Traffic	Urban Environment	Number of vehicles circulating per km^2 of urban area.	Istat	urban_traffic
Number of Vehicles	Urban Environment	Number of vehicles circulating per km^2 of territorial area.	Istat	vehicles_total
Green Areas	Urban Environment	Density of urban green areas (%).	Istat	green_urban
Electricity Consumption	Urban Environment	Total electricity consumption (GWh) required of the distribution network.	Istat	Electric_consumption

Power of Photovoltaic Solar Panels	Urban Environment	Total power of photovoltaic solar panels (kW per 1000 inhabitants).	Istat	Photovoltaic_panel
*Index of non-achievement of the first cycle school**	Socio-economic Data	Percentage of students who did not achieve the first cycle school out of the total (%).	Istat	instruction
Life Quality	Socio-economic Data	Calculated by examining 90 indicators, divided into six thematic macro-categories, since 1990.	Il Sole 24 Ore	life_quality
Expectation of Life	Socio-economic Data	Age of life expectancy.	Istat	lifetime
*Number of Beds in Hospitals**	Socio-economic Data	Number of beds available in hospitals.	Istat	bed_number
*Income**	Socio-economic Data	Average gross taxable income for each individual (euro).	Istat	income
Circulatory System	Other Pathologies	Mortality rate due to diseases related to *circulatory system*.	Istat	circulatory_mort
Digestive System	Other Pathologies	Mortality rate due to diseases related to *Digestive System*.	Istat	digestive_mort
Brain Tumors	Other Pathologies	Mortality rate due to diseases related to *Brain Tumors*.	Istat	brain_mort
Ischemia	Other Pathologies	Mortality rate due to diseases related to *Ischemia*.	Istat	ischemia_mort

Diabetes	Other Pathologies	Mortality rate due to diseases related to *Diabetes*.	Istat	diabetes_mort

Table 1: Data used over the analysis. *Data at municipal level, then grouped into provinces.

Shap Values

	**2015**	**2016**	**2017**	**2018**	**2019**
O3	0.107	0.040	0.056	0.055	0.020
NO2	0.021	0.069	0.021	0.038	0.070
SO2					0.034
CO			0.032		
PM10			0.018		0.024
Benzene		0.047			0.021
AQI	0.017			0.020	
P4O10 fertilizer		0.024	0.017	0.018	0.015
Microelement fertilizer				0.025	
Organic fertilizer			0.039		
N fertilizer			0.038	0.021	0.015
Cultivated Areas	0.028				
lifetime	0.013				
Life quality		0.012			
instruction		0.027			
income	0.017				
Electric consumption	0.015				
Photovoltaic panel	0.023				
Diabetes mort		0.014			0.015
Circulatory mort	0.020	0.036	0.024	0.039	0.012
Digestive mort	0.020		0.026	0.021	0.035
Ischemia mort	0.025	0.019		0.030	

Table.2 Mean of the absolute value of Shapley values. The sum of each Shapley values makes the final prediction of the model. The blank cells refer to unselected feature by Boruta algorithm.
